# Design of a Smart IoT-Based Control System for Remotely Managing Cold Storage Facilities

**DOI:** 10.3390/s22134680

**Published:** 2022-06-21

**Authors:** Maged Mohammed, Khaled Riad, Nashi Alqahtani

**Affiliations:** 1 Date Palm Research Center of Excellence, King Faisal University, Al-Ahsa 31982, Saudi Arabia; nalqahtani@kfu.edu.sa; 2 Department of Agricultural and Biosystems Engineering, Faculty of Agriculture, Menoufia University, Shebin El Koum 32514, Egypt; 3 Department of Computer Science, College of Computer Sciences and Information Technology, King Faisal University, Al-Ahsa 31982, Saudi Arabia; kriad@kfu.edu.sa; 4 Department of Mathematics, Faculty of Science, Zagazig University, Zagazig 44519, Egypt; 5 Department of Food and Nutrition Sciences, College of Agricultural and Food Sciences, King Faisal University, P.O. Box 420, Al-Ahsa 31982, Saudi Arabia

**Keywords:** internet of things (IoT), sensors, microcontroller, monitoring, micro-climate, control, ThingSpeak, ESP8266, Arduino, date fruit quality, food preservation, energy, electrical power

## Abstract

Cold storage is deemed one of the main elements in food safety management to maintain food quality. The temperature, relative humidity (RH), and air quality in cold storage rooms (CSRs) should be carefully controlled to ensure food quality and safety during cold storage. In addition, the components of CSR are exposed to risks caused by the electric current, high temperature surrounding the compressor of the condensing unit, snow and ice accumulation on the evaporator coils, and refrigerant gas leakage. These parameters affect the stored product quality, and the real-time sending of warnings is very important for early preemptive actionability against the risks that may cause damage to the components of the cold storage rooms. The IoT-based control (IoT-BC) with multipurpose sensors in food technologies presents solutions for postharvest quality management of fruits during cold storage. Therefore, this study aimed to design and evaluate a IoT-BC system to remotely control, risk alert, and monitor the microclimate parameters, i.e., RH, temperature, CO_2_, C_2_H_4_, and light and some operating parameters, i.e., the temperature of the refrigeration compressor, the electrical current, and the energy consumption for a modified CSR (MCSR). In addition, the impacts of the designed IoT-BC system on date fruit quality during cold storage were investigated compared with a traditional CSR (TCSR) as a case study. The results showed that the designed IoT-BC system precisely controlled the MCSR, provided reliable data about the interior microclimate atmosphere, applied electrical current and energy consumption of the MCSR, and sent the necessary alerts in case of an emergency based on real-time data analytics. There was no significant effect of the storage time on the most important quality attributes for stored date fruit in the MCSR compared with the TCSR. As a result, the MCSR maintained high-quality attributes of date fruits during cold storage. Based on the positive impact of the designed IoT-BC system on the MCSR and stored fruit quality, this modification seems quite suitable for remotely managing cold storage facilities.

## 1. Introduction

Date fruit (*Phoenix dactylifera* L.) is cultivated on a 1.25 million hectares area of the world with an annual production of 9.61 Mt, whereas in Saudi Arabia, its cultivated area is 0.15 million hectares producing a 1.54 Mt yield [1]. Most fruits of date palm cultivars are consumed at the fully ripen stage, known as the Tamr stage [2,3]. The stored fruit products are usually accompanied by respiration, evaporation, and physiological changes due to moisture losses, which could induce heavy losses reaching up to 40%. Therefore, suitable storage facilities are essential for extending the shelf life of date fruits [4]. On the other hand, cold storage systems maintain the quality of agricultural products by reducing moisture loss and slowing physiological changes [5]. Therefore, cold storage techniques are used widely for perishable commodities such as fruits and vegetables to retain their shelf life after harvest [6,7]. In addition, it prevents the growth of contaminating microorganisms and reduces the rate of biochemical changes in fresh produce [8,9]. The increased use of cold storage facilities is due to the year-round availability of fresh produce in the market. Moreover, the surfeit supply of fruits and vegetables in the market has a negative impact on the income of the growers. Therefore, surplus fruits and vegetables are stored to control price fluctuations and maintain the commodity’s availability in the market [10]. Food type, general appearance, including any cosmetic damage, bruises, or microbial contamination, the maturity stage, and the storage room temperature and RH are all factors that influence the shelf life of fresh fruits in cold storage [11,12].

In the storage of fresh fruits, the temperature is closely correlated to respirational rates. The respirational rates can be reduced by reducing the temperature to a particular limit depending on the stored products. The lower respiration rates decrease the kinetics of biochemical responses including those related to product quality [13]. In addition, the temperature is an important parameter that affects the life of the food product by directly impacting the rates of biochemical activities [14]. Through the process of respiration and microbial degradation, the nutrients of the product are broken down into simpler compounds which often cause the quantity and quality reduction in the foods [5]. All these mentioned processes are highly dependent upon the storage temperature.

Fruits are stored at non-freezing temperatures to preserve tissue cells and prevent cell structure disintegration and postharvest damage [15]. Therefore, fruits of the date palm harvested at the Tamer stage are normally kept in non-freezing storage chambers to prolong their shelf-life [16]. Refrigeration or cold storage is the most common method used to increase the shelf life of dates [17]. Fruit weight loss and microbial degradation, such as yeast and bacterial fermentation and postharvest fungal infections, are the most significant factors restricting date fruit storage [18]. Therefore, temperature and RH should be carefully considered when storing date palm fruits to extend their postharvest life and commercialization period [9]. Low-temperature storage also protects non-aesthetic quality characteristics of fruits, such as texture, nutrition, aroma, and flavor [19]. Date fruits need to be kept at low temperatures immediately after harvesting to slow down the growth of microorganisms and insect activity responsible for quality deterioration. In addition, low temperature minimizes the vapor pressure between the outer atmosphere and the product, reducing the water loss from the fruits [20].

The modern multi-target concept of food preservation is becoming more attractive with recent technologies to maintain their quality during storage [9,21]. The fruit preservation is presently a multidisciplinary science to best disease control with the desired physicochemical characteristics for quality maintenance as long as possible [22,23]. The Internet of things (IoT) facilitates real-time monitoring of the system’s environment in many areas. It allows the systems to respond suitably and timely [24]. The applications of IoT-Based multipurpose sensors for food technologies have paved the way for intelligent food quality management. These integrated technologies can give reliable data on the quality of food products during their storage duration [25]. IoT in cold storage aims to monitor parameters that affect the stored product quality and preserve it from contamination due to surrounding conditions. The most important parameters are RH, temperature, alcohol gases, and light in cold storage rooms and warehouses [26,27].

There are some challenges for the cold storage monitoring systems, such as supporting distinct requirements for various products, tracking products and labeling, real-time monitoring, minimizing human intervention, and monitoring of various parameters. There are various attempts to integrate IoT with cold storage, and each of them has its own contribution. Afreen and Bajwa [28] presented a real-time intelligent monitoring and notification system (RT-IMNS) based on IoT to introduce real-time monitoring of important parameters such as temperature, luminosity, humidity, and gas concentration in cold storage. The RT-IMNS can send notifications when exceeding some dangerous limits for the measured parameters. Appasani et al. [29] proposed an advanced automated cold storage system (ACSS). The authors monitored the temperature and humidity inside the cold storage room. They considered these two parameters the most important for any cold storage system. They introduced self-adaptable capabilities using a controller using a particle swarm optimization algorithm. Sarmah and Aruna [30] used heterogeneous IoT devices and cloud services to track food quantity. The model accurately detects the food quality and sends an alert in case of degradation. Umamaheswari et al. [31] proposed smart cold storage that speeds up the supply chain. The scheme employed Raspberry Pi-3 B+ in combination with some sensors. This scheme can detect and identify the objects inside the smart cold storage using a web camera. Additionally, a load cell with HX711 IC have been used to weight the objects inside the smart cold storage. Feng et al. [32] introduced an evaluation of an IoT-based monitoring system (IoTMS) for cold storage. The IoTMS introduced an electronic nose to detect food spoilage. The authors used this electronic nose to detect some parameters for the salmon samples. This scheme also used the convolutional neural networks and support vector machine to classify the levels of freshness. Srivatsa et al. [33] were motivated by the need for perishable goods to travel thousands of miles until they reached their destination. They introduced an endless real-time monitoring system using IoT that controls the losses which occur for businesses for 7645 storage facilities in India.

The efficient monitoring and control of the temperature and RH in the internal atmosphere of cold storage rooms leads to preserving date quality and reducing water loss during storage [9]. Ganjewar et al. [34] protected the food from getting spoiled by introducing real-time monitoring and control system. They employed IoT and adaptive Naïve Bayes prediction to preserve the food from being spoiled. They collected the data from the IoT sensors and executed projections on it. This scheme is interested in environmental parameters such as temperature, moisture, RH, and light. Yadav et al. [27] used Raspberry pi as a monitoring node with python’s programming language. The Raspberry Pi board is interfaced with multi-sensors of MQ3 to determine alcohol contents, LDR to determine the light intensity, and DHT11 to measure the temperature and RH. Mishra et al. [35] developed a low-cost solar-powered smart cold storage. They used a modified split air conditioner for cold storage. This low-cost cold storage supports automatic remote monitoring using IoT sensors. Zhan et al. [36] employed industrial IoT and unsupervised learning to develop an intelligent real-time occupational safety monitoring system for cold storage. They used a stacked auto-encoder to track abnormal stationary detection in cold storage. Additionally, they provided enhanced spatial-temporal traceability by using some intelligent services. Finally, they introduced real-life air cargo cold storage as a case study. Gautham et al. [37] employed machine learning and IoT to build an ultrasonic humidification control system to extend the longevity of fruits and vegetables. In this scheme, the IoT provides visual feedback for the current levels for some parameters of interest.

In the previous studies, they only monitored the microclimate atmosphere of CSRs using the IoT regarding temperature and humidity. However, the microclimate atmosphere of CSRs consists of other gases that must be monitored, such as CO_2_ and C_2_H_4_, to maintain the quality of stored products and the health of workers. Moreover, if the main door of the CSR is left open it causes outside air to flow inside the CSR which changes the temperature, relative humidity, and gas concentrations inside the CSR and increases the power consumption. Previous studies also have shown that the humidification of CSRs using ultrasonic humidifiers has the possibility to improve the quality of the stored product. Real-time remote control and malfunction alerts for the microclimate atmosphere of CSRs and their equipment, i.e., evaporator unit, condensing unit, control unit, humidification unit, and defrost unit are needed to maintain the CSR and quality of stored products. So, when sudden malfunctions occur in the CSR equipment and the control system fails to solve the problem or the user leaves the light or door open for more than the predefined Setpoints time the IoT system should give notification with an alarm for maintaining food quality and energy saving. In addition, compressor temperatures and electrical energy consumption must be carefully monitored and controlled so that appropriate actions can be taken in real-time. Accordingly, there is still a need to design a modern, low-cost, viable system for controlling, providing visual feedback on the current data for CSR microclimate and its equipment, and sending warning alerts for reducing post-harvest losses and storage issues.

Therefore, this study aimed to design and implement a smart IoT-BC system to remotely control, alert, and real-time monitor the interior microclimate, i.e., relative humidity, temperature, CO_2_, C_2_H_4_, and light and some operating parameters, i.e., the temperature of the refrigeration compressor, the electrical current, and the energy consumption of a CSR to extend shelf life and maintain high-quality attributes of stored fruit and support components of cold storage facilities. In addition, evaluating the designed IoT-BC system regarding its performance, operating the modified CSR, and its impact on stored date fruit quality compared to the traditional CSR.

## 2. Materials and Methods

### 2.1. Cold Storage Rooms Description

**Figure 1:**
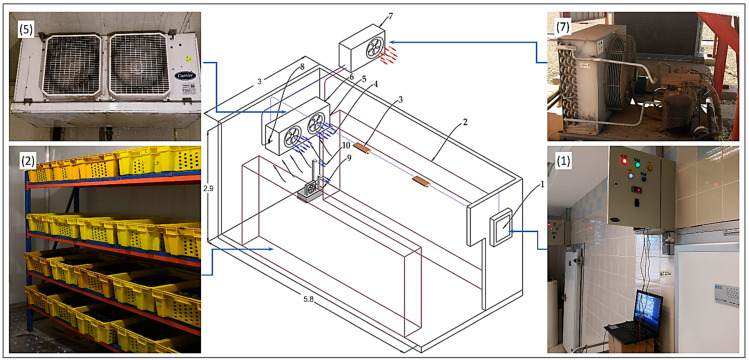
The main component of the cold storage room (dimensions in m). (1) Control unit, (2) storage shelves, (3) LED light bulbs, (4) cold air direction, (5) evaporator unit, (6) electrically operated expansion device, (7) condensing unit, (8) pressure transmitter, (9) ultrasonic humidifier, (10) outputted mist (all dimensions in meters).

The experiment was conducted using two similar cold storage rooms CSRs established at the Date Palm Research Center of Excellence (DPRC), King Faisal University (KFU), Saudi Arabia. One of these CSRs was remotely controlled and monitored by the designed cloud-based IoT system (IoT-BC), and the second was left with its traditional control. Figure 1 shows the main components of the CSR, which consisted of a thermally insulated cold room, evaporator unit, condensing unit, control unit, humidification unit, and defrost unit. The internal measures of the cold room were 5.8 m in length, 2.9 m in width, and 2.9 m in height.

The CSR walls and ceiling were made from polyurethane sandwich panels (PUSP) with 0.12 thickness. The PUSP was composed of three layers. The internal and external layers were two corrugated aluminum plates with a thickness of 0.01 m. The core layer was high-density polyurethane foam with a thickness of 0.10 m. The evaporator (type: EVAP.CAE3264 R417A, Carrier s.c.s. Co., Aubagne, France) is equipped with two fans 230 V/single-phase/60 Hz. The R417 A refrigerant (Type: CAE3264, −40/38 °C min/max) was used in the cooling system of the CSR. The condensing unit is equipped with a refrigeration compressor 3 phase, 380–420 V/3-phase/60 Hz, and the nominal operating current was 6.8 A (model DLEE-201-EWK, Emerson Climate Technologies Ltd., Mikulov, Czech Republic). The control unit consisted of one 3-phase breaker, two single-phase breakers, three contactors, a 24 h timer, and a digital temperature controller 230 VAC (model: XR06CX, Dixell, Pieve d’Alpago, Italy). The control unit was used to control the operating fans and CSR compressor. The CSR included three compact fluorescent light bulbs with a total power of 75 W. The temperature inside the CSR was set at the target temperatures before conducting the experiments using the controller supplied in the control unit of each CSR.

The humidification unit was an ultrasonic humidifier with the same descriptions as the designed ultrasonic humidifier by Mohammed et al. [9]. The ultrasonic humidifier was used to control the relative humidity (RH) in the CSR. This humidifier consisted of ten ultrasonic transducers, a water reservoir, an air fan, and a control unit. The ultrasonic transducers were installed at the central bottom position of the reservoir. The frequency of each ultrasonic transducer was 2600 kHz with a resonance impedance of 2 Ω, and the operating temperature range was 0 to 50 °C. The humidifier reservoir was a rectangular stainless-steel tank of 0.25 × 0.25 × 0.40 m. The fan flow rate ranged from 0 to 2.5 m^3^/min with a maximum power consumption of 22 W, and the rated inputted voltage was 230 V/60 Hz. The RH was adjusted by the high-precision humidity control unit (MH13001 with a humidity sensor of INS121, Shenzhen Electronic Co., Ltd., Shenzhen, Guangdong, China).

The accumulated frost on the surface of the evaporator coils hinders the distribution and conduction of the cooling capacity of the refrigeration evaporator, which finally impacts the cooling efficiency or deforms the evaporator. This problem leads to increasing electrical energy consumption and reducing the service life of refrigeration system equipment. Therefore, the benefit of the defrost unit is to defrost the accumulated frost to improve the efficiency of the system cooling, ensuring the quality of stored products in the storage room, saving electrical energy, and extending the service life of the cold storage components. Therefore, the electric heating type (220 V, 200 W) was used for the defrost unit CSRs. The heaters of the defrost unit were clamped directly to the evaporator coil and operated by the control based on defrosting temperature sensor data to prevent the coils from damaging by freezing water.

### 2.2. The IoT-Based Control System Architecture

**Figure 2:**
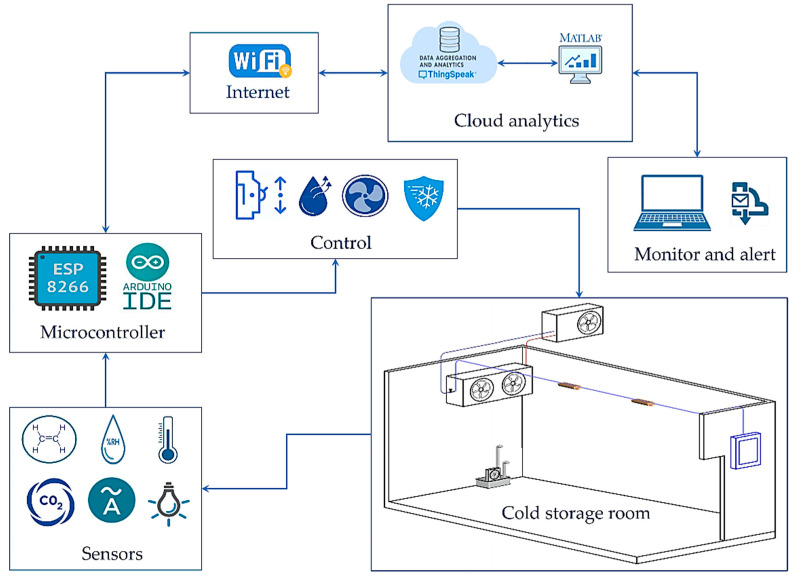
The system model of the designed IoT-BC.

The detailed workflow and communication among the different entities of the designed IoT-based control system (IoT-BC) are shown in Figure 2. The figure shows the modified cold storage room (MCSR) that was monitored and controlled remotely. In addition, a set of sensors and microcontrollers for collecting data were used to control and monitor the selected parameters. The communication between the different entities is as follows:

- The sensors periodically collected the data from different portions of the cold storage room.
- The collected data were uploaded by the considered microcontrollers to our private channel on ThingSpeak through Wi-Fi for control, monitoring, and alert purposes.
- The data analytics was accomplished on our ThingSpeak channel using MATLAB Analysis App. According to the code written on MATLAB Analysis App, the desired action/control is sent in real-time to the designated microcontroller to be applied to the cold storage room.
- The authenticated users can access our private ThingSpeak channel and monitor real-time through graphical interfaces to all the measured parameters.
- Finally, two alerts (email and SMS) are sent to a designated administrator in case of an emergency based on real-time data analytics. The email alert was done using the MATLAB Analysis App. The SMS alert was accomplished using the ThingHTTP App and our account on the Twilio website.

The main goal of the designed IoT-BC is monitoring some critical parameters, i.e., the atmosphere storage, controlling its work, and sending alerts in cases of emergency. Therefore, the IoT-BC monitored the internal and external atmosphere temperature, internal relative humidity (RH), Internal Carbon Dioxide concentration (CO_2_), internal ethylene gas concentration (C_2_H_4_), internal ambient light, compressor temperature, electrical current intensity, and electrical energy consumption.

The IoT-BC controlled the humidification unit based on the relative humidity levels, the ventilation unit based on the CO_2_ and C_2_H_4_ levels, and the control unit based on the internal atmosphere temperature, compressor temperature, and current intensity.

The alerting email and SMS were achieved for the following parameters:

- Internal temperature measured by the internal DHT22 sensor;
- Internal relative humidity measured by the internal DHT22 sensor;
- Internal carbon dioxide CO_2_ measured by the Senseair-S8 sensor;
- Internal ethylene gas C_2_H_4_ measured by the MQ-3 sensor;
- Internal ambient light measured by the BH1750 sensor;
- Compressor temperature measured by the DS1822-PAR sensor;
- The applied current intensity measured by three current meters.

#### 2.2.1. IoT-BC Hardware Layout

**Figure 3:**
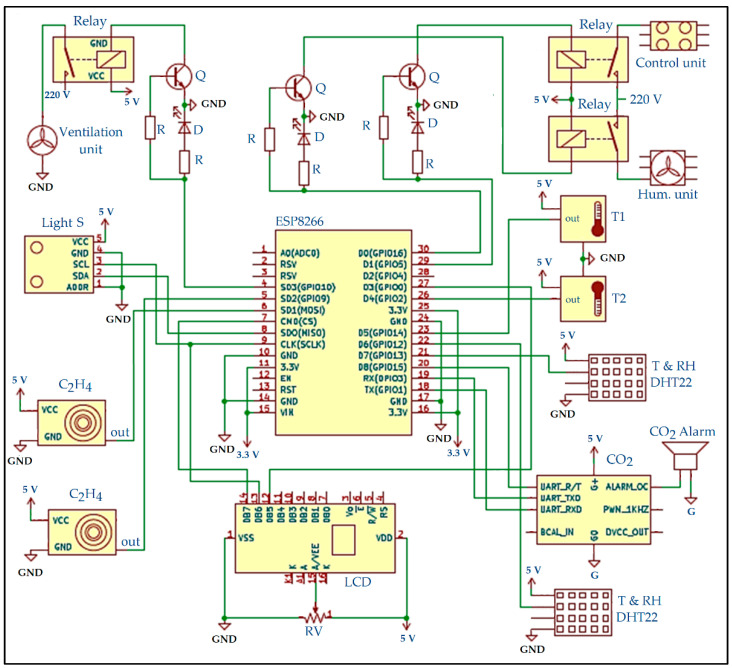
The detailed schematic KiCad diagram with successful electrical rules check for the internal sensors. D is a diode, ESP8266 is a NodeMCU board, GND is the ground, Q is transistor (BC547), R is a resistor, RV rheostat, T1 and T2 are temperature sensors (DS1822), T&RH is temperature and relative humidity sensor (DHT22), LCD is a liquid crystal display (RC1602A-GHW-ESX), S is sensor and Hum is humidification.

This section introduces the schematic diagrams in IoT-BC, the description of the used sensors, and the calibration of the sensor. The hardware layout has been implemented using KiCad version 6.0 installed on MacBook Pro (3.3 GHz Intel Core i7) to draw the expected IoT-BC schematic and confirm the electrical rules check, as shown in Figure 3. KiCad has been used for its ability to handle and maintain various and complex electronic circuits. For example, two circuits were used to monitor and control the controlled atmosphere storage: one circuit for the internal atmosphere storage, and the second circuit for the compressor operations.

Figure 1 introduces the detailed KiCad diagram for the set of considered sensors after running an electrical rules check. As shown in this figure, the description part consists of the following items:

- Comments related to the connected peripherals:
  Comment 1: The CO_2_-Alarm beeps when the CO_2_ value reaches 0.045%.
  Comment 2: GPIO 5 is the output for the internal temperature measured by the DHT22.
  Comment 3: GPIO 10 is the output for the ventilation unit based on the CO_2_ and Alcohol.
  Comment 4: GPIO 16 is the output for the humidification based on the humidity level.

The details of the connected peripherals in our IoT-BC are as follows:

- ESP8266 NodeMCU board: We used one ESP8266 NodeMCU board. It is used to interface the IoT-BC peripherals with the Internet and the cloud platform. Several 24 pins have been used to connect the ESP8266 with the IoT-BC sensors and actuators. There are six empty pins between power and multiplexed GPIO pins that can be used for future scaling purposes using the same board.
- DS1822-PAR Sensor: It is a single-wire distributed temperature sensor. It is based on the multidrop capability that simplifies distributed temperature sensing applications. Therefore, it does not need any external components. Its measurement range is from −55 ± 100 °C. Two DS1822-PAR sensors have been employed in the IoT-BC. They have been connected to GPIO2 and GPIO14.
- DHT22 Sensor: It is a low-cost digital temperature and relative humidity sensor. Its temperature range is −40–80 °C. Its RH range is 0–100%. It reads a new measure every 2 s. Two DHT22 have been employed in the IoT-BC. They have been connected to GPIO12 and GPIO13.
- Senseair-S8 (004-0-0075) Sensor: It is a commercial miniature infrared CO_2_ sensor. Its measurement range is 400–2000 ppm. It reads a new measure every 2 s. It is equipped with two output pins—an alarm output and a 1 kHz PWM output. One Senseair-S8 sensor has been employed in the IoT-BC. It has been connected to the UART pins (GPIO1 and GPIO3) and one digital pin (GPIO5).
- BH1750 Sensor: It is a digital ambient light sensor. It has been interfaced with the ESP8266 module using the I2C serial interface. It has been connected to MOSI and SCLK pins. It is used to sense the light inside the CCSA.
- MQ-3 Sensor: It is a sensitive gas sensor. It is used in IoT-BC to detect the existence of ethylene gas (C_2_H_4_). Once the ethylene alcohol gas exists, the sensor’s conductivity gets higher, and the gas concentration rises. Two MQ-3 sensors have been used in IoT-BC. They have been connected with the ESP8266 module through GPIO9 and GPIO10.
- G5Q-1A Electrical Relay: Three G5Q-1A electrical relays have been used in IoT-BC. They are connected to GPIO4, GPIO5, and GPIO16. The first relay (connected to GPIO5) controls the temperature inside the IoT-BC. The second relay (connected to GPIO16) controls the humidification unit in the IoT-BC. Finally, the third relay (connected to GPIO4) controls the ventilation unit in the IoT-BC.
- RC1602A-GHW-ESX LCD: It is the only LCD used in IoT-BC. It is mainly used to display some information related to doing action on a real-time basis. It has been connected with the ESP8266 module through CS, SCLK, and GPIO0.

**Figure 4:**
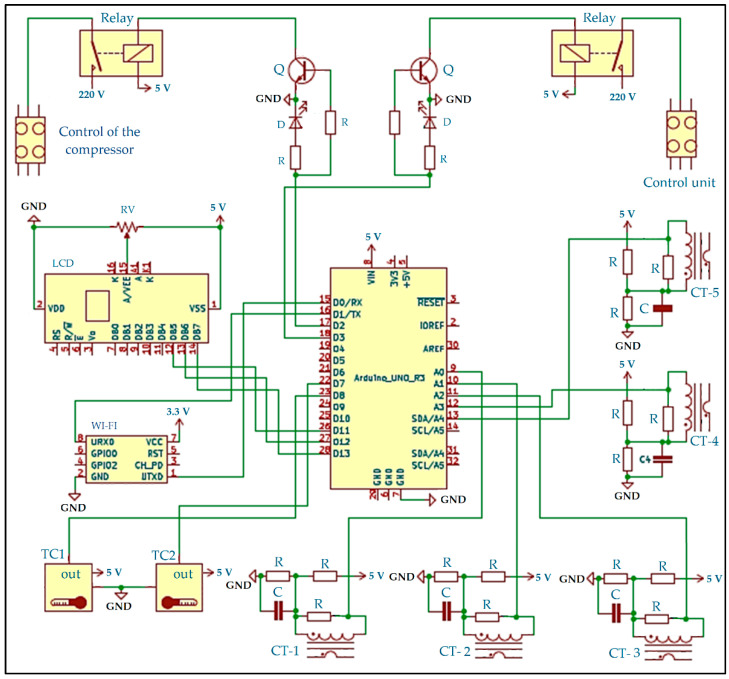
The detailed schematic KiCad diagram with successful electrical rules checks for the current meters and power control. C is a capacitor, D is a diode, GND is the ground, Q is transistor (BC547), R is a resistor, RV rheostat, TC1 and TC2 are temperature sensors of the compressor (DS1822), LCD is a liquid crystal display (RC1602A-GHW-ESX), CT-1, CT-2 and CT-3 is the current meter of the compressor, CT-4 is the current meter of the condensing unit, CT-5 is the current meter of the control unit, WI-FI is a Wi-Fi module (ESP-01090).

Figure 4 introduces the detailed KiCad diagram for the set of considered current meters and sensors after running electrical rules check for all of them. As shown in this figure, the description part consists of the following items:

- Comments related to the connected peripherals:
- Comment 1: A current transformer is used with some resistances to measure the current.
- Comment 2: Three current meters are used for the compressor.
- Comment 3: Two current meters are used for the condensing and control unit.
- The number of sheets and the file name (Cloud_CAS_2.kicad_sch).
- The title of this schematic diagram (Cloud-Controlled Atmosphere Storage (IoT-BC)—Current Meter).

Moreover, the details of the connected peripherals in the current control schematic are as follows:

- Arduino UNO R3: It is a microcontroller board based on ATmega328P. It provides 14 digital input/output pins and six analog pins.
- ESP-01v090: It is a Wi-Fi module from ESPressif. It connects the Arduino board to the Internet. It is interfaced with the Arduino board through Tx and Rx pins.
- Current meter: The employed current meter comprises one current transformer, one capacitor, one 33-ohm burden resistor, and two 10–470 K-ohm potentiometers. Five current meters have been connected to the Arduino UNO R3 board. They are connected to the following analog pins A0, A1, A2, A3, and A4.
- DS1822-PAR Sensor: Two DS1822-PAR sensors have been employed in the current control schematic. They have been connected to D7 and D8.

#### 2.2.2. IoT-BC Software Layout

**Figure 5:**
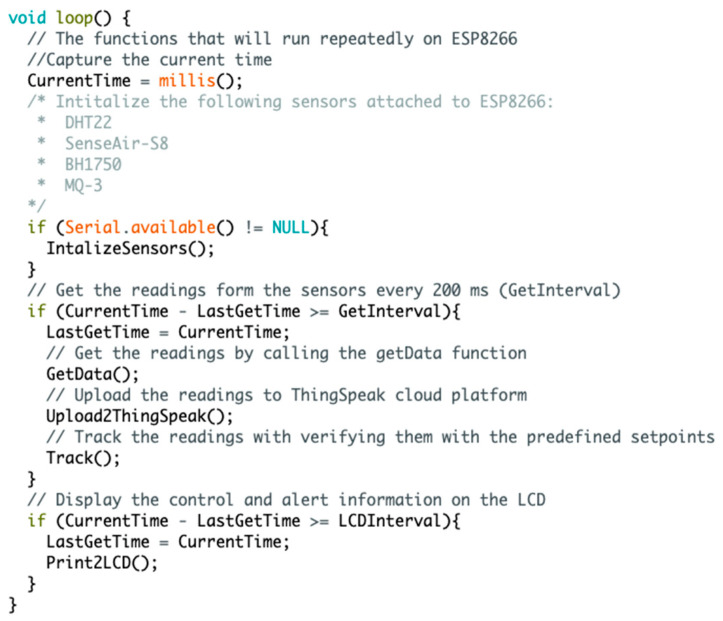
The repeatedly executed code on the ESP8266 board.

There are two codes used in the IoT-BC. The first one is used to monitor and control/alert the internal atmosphere storage (Figure 3), and the second one is used to monitor and control/alert the current (Figure 4). For simplicity reasons, only the code used for controlling the internal atmosphere storage is introduced here, as shown in Figure 5.

The figure shows the code that will be repeatedly executed on the ESP8266 board to monitor, control, and alert some of the main parameters considered in IoT-BC. The detailed description of the main functions used in this code is as follows:

- IntializeSensors( ): This function is responsible for executing sensors’ calibration and reset if needed. Each sensor is assigned a unique name in the introduced internal control to the atmosphere storage. This unique name helps send instructions to the sensors and collect the data from them.
- GetData( ): The readings will be collected from the sensors every 200 ms. This reading interval is represented by the if condition “if (CurrentTime—LastGetTime >= GetInterval)”. Thus, every 200 ms, the GetData( ) collects the readings from the list of sensors attached to the microcontroller. This process is accomplished based on each sensor and its own configuration.
- Upload2ThingSpeak( ): This function is mainly used to send the collected reading from the list of sensors to a private cloud channel on the ThingSpeak platform. This upload stage is done in real-time. The real-time measures uploaded to the cloud ThingSpeak platform are used for data analytics and sending alerts to a designated administrator in case of emergency based on each measured parameter.
- Track( ): This function is considered local monitoring that shows the system activity. All the readings collected from the sensors are displayed on the serial monitor at the collection time. Additionally, in case of an emergency, the alert message is shown multiple times on the serial monitor to alert the local investigator about the emergency issue.
- Print2LCD( ): This function execute a similar task to that of Track( ) function. However, this function will show all the readings and alerts on the LCD.

### 2.3. Sensors Calibration

The Food and Drug Administration (FDA) requires an accurate display for all devices that have an impact on the quality of the stored product by their calibrating over a specified interval of time. The equipment calibration is also important to facilitate the storage of many critical materials that demand a controlled modified atmosphere and security. In order to comply with the international benchmarks, tested and calibrated CSR rooms (including their cooling equipment) according to the Code of Federal Regulations Title 21 were used in this study [38]. The sensors used in IoT-BC have been calibrated to assure their accuracy as follows:

- To calibrate the temperature and humidity sensor DHT22 and the digital temperature sensors, a controlled atmosphere (temperature and RH) incubator (model: PC900h, Helmer Scientific Inc., Noblesville, IN, USA) was used. The incubator atmosphere temperatures and RH were set at different values and the observed values were compared with the values of the sensor’s readings.
- To calibrate the CO_2_ sensor, the reading of the Senseair-S8 sensor was compared with the reading recorded by the indoor air CO_2_ m/datalogger (model: Extech EA80, FLIR Commercial Systems Inc., Nashua, NH, USA) at a temperature of 5 C. A carbon dioxide cylinder containing 99.5% pure CO_2_ was used to add different concentrations of CO_2_ in a closed room.
- To calibrate the ethylene MQ-3 sensor, a fruit ripening gas box containing 99.95% pure ethylene (type: corrugated box of 96 Pc, MILAN JYOTI INTERNATIONAL LTD, Mumbai, India) was used. The gas was sprayed in a closed cold room at a temperature of 5 °C and the reading of the sensor was compared with the reading of a portable ethylene gas detector (model: PG-100-C_2_H_4_, AMBETRONICS ENGINEERS PVT LTD., Mumbai, India).
- To calibrate the light intensity BH1750 sensor, the reading of the sensor was compared with the reading recorded by the light intensity meter/datalogger (model: Extech EA33, FLIR Commercial Systems Inc., Nashua, NH, USA). Calibration was carried out using the LED light source with variable intensity of illumination in the cooling room at a temperature of 5 °C.
- To calibrate the current intensity Load_CellX03 with HX711 amplifier, power, and electrical energy consumption, the reading of the current sensor, calculated power, and calculated energy consumption were compared with the reading recorded by three-phase power and harmonics clamp meter (UNI-T, UT243, Uni-Trend Technology Co., Ltd. Dongguan, Guangdong, China) under different current intensities consumed by a heater coil.

### 2.4. Control and Alert

IoT-BC’s control and the alert system have been introduced to control the compressor, the ventilation unit, and the humidification unit. As well as sending alerts to a designated administrator based on the measures of some sensors. Moreover, sending an alert based on the light level inside the atmosphere storage. In comparison to the Supervisory Control and Data Acquisition (SCADA) system which is very similar to the designed IoT-BC. The SCADA system could remotely control huge industrial processes in real-time based on high-level software applications. Additionally, a trained user is needed to use the SCAD system. Thus, the SCADA system is most suitable for the high cost and huge industrial facilities, as it is very expensive. The SCADA system also needs huge computational resources to work properly. On the other hand, the designed IoT-BC could achieve the same objectives as the SCADA system with low cost for the small to medium-sized industrial facilities. Moreover, the designed IoT-BC requires a simple IoT microcontroller and sensors along with a free cloud platform. Additionally, the normal user can use IoT-BC’s interface as it is very simple and easy to use.

**Figure 6:**
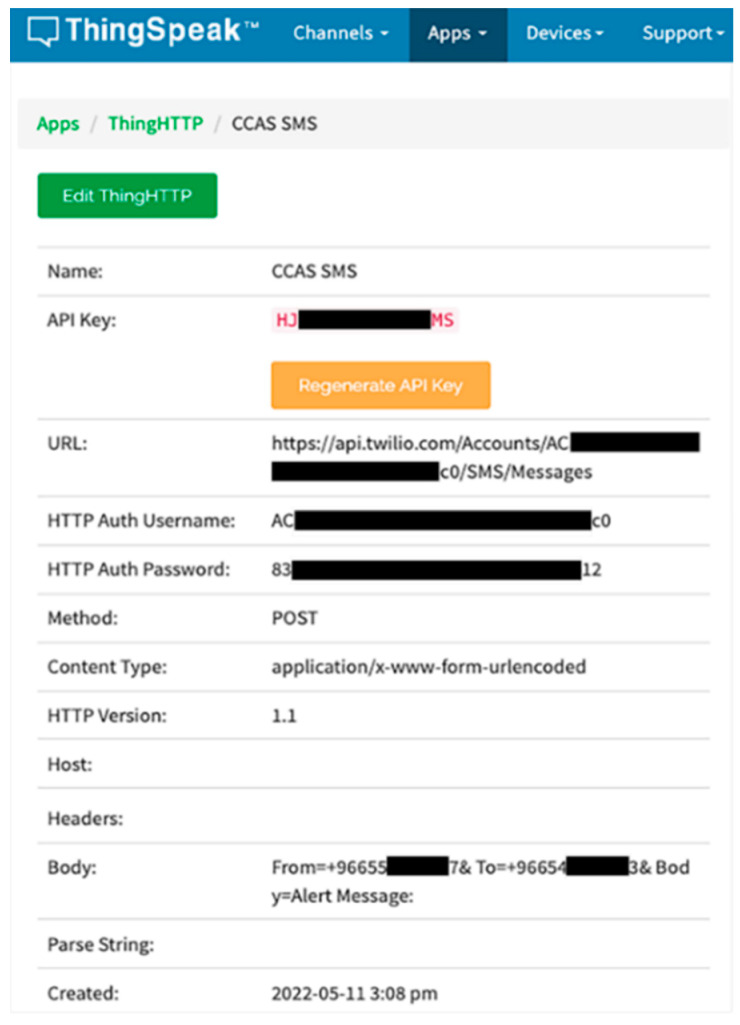
The detailed configuration for using ThingHTTP and Twilio website to send an alert SMS to a designated administrator.

The alert in IoT-BC is an email and SMS for a designated administrator through our private channel on the ThingSpeak cloud platform. The configuration for sending an email is achieved using MATLAB Analysis App on ThingSpeak. Where a MATLAB code is written in the MTLAB Analysis App for each controlled parameter. Additionally, the configuration of sending an SMS using ThingSpeak and the Twilio website is shown in Figure 6. The figure shows the detailed configuration for sending an SMS as an alert by using the Twilio website and ThingHTTP App on our private channel on the ThingSpeak cloud platform.

#### 2.4.1. Compressor Control and Alert Mechanism

**Figure 7:**
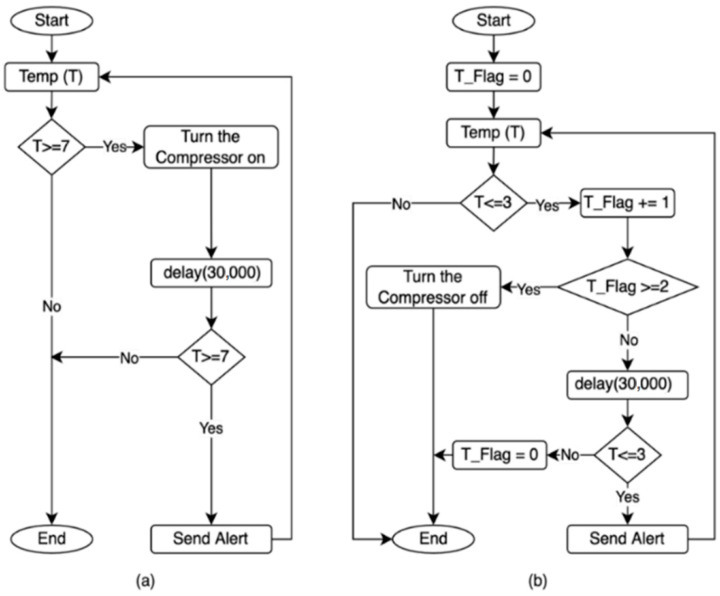
The compressor control and alert based on the internal temperature (T) measured by the DHT22 sensor. (**a**) The control and alert based on the maximum setpoint. (**b**). The control and alert based on the minimum setpoint.

**Figure 8:**
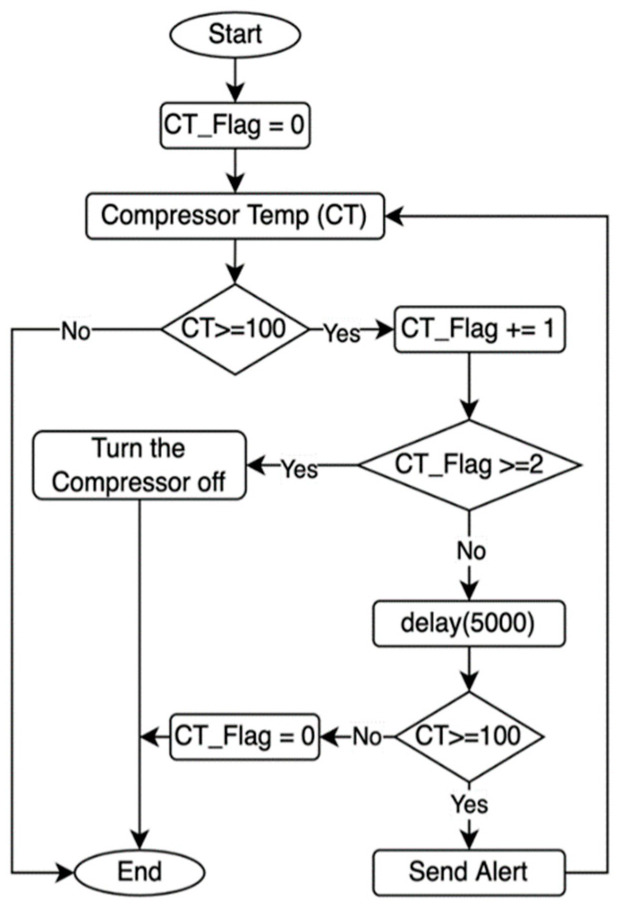
The compressor control and alert based on the compressor temperature (CT) measured by the DS1822-PAR sensor.

**Figure 9:**
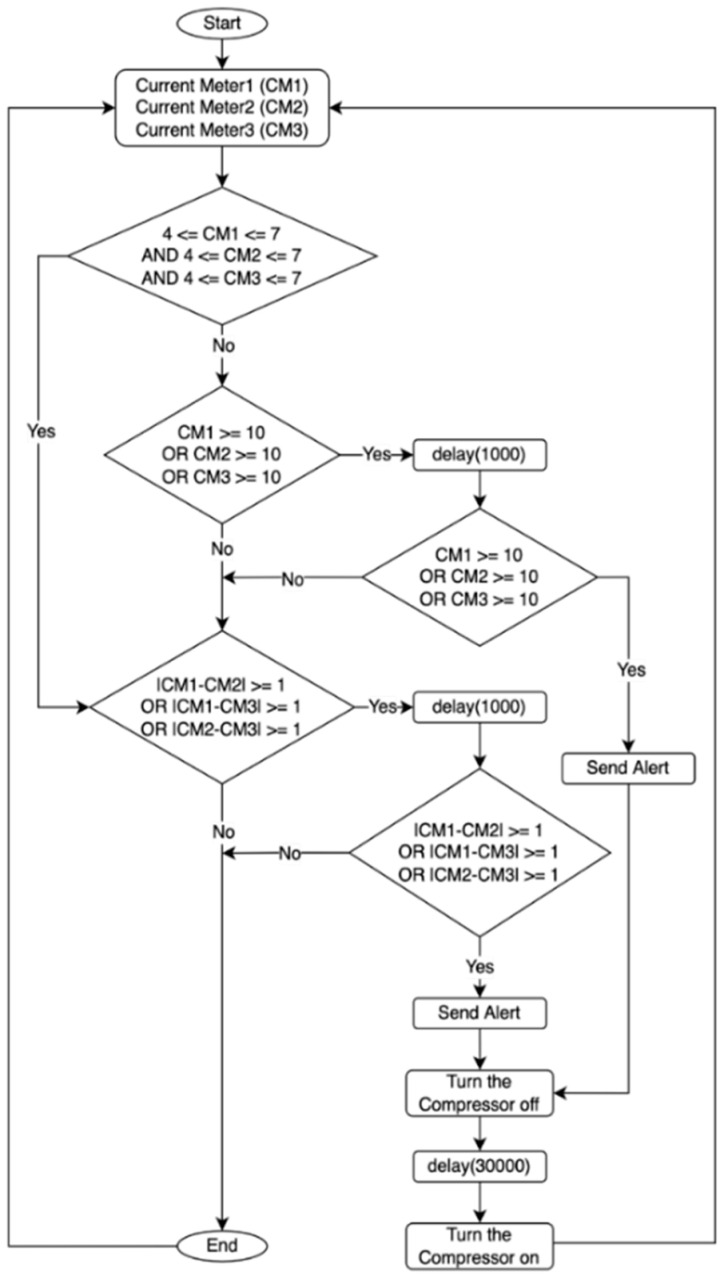
The compressor control and alert based on three current meters measures (CM1, CM2, and CM3).

The process of controlling the compressor in the MCSR has been accomplished based on three main parameters:

- Internal Temperature (T): It is shown in Figure 7. The flowchart (Figure 7a) turns the compressor on when the internal temperature reaches the maximum setpoint (T is greater than or equal 7 °C). The IoT-BC will send an email and SMS to a designated administrator to alert if the internal temperature remains greater than the maximum temperature set point for 30 min after turning the compressor on. The flowchart (Figure 7b) turns the compressor off when the internal temperature reaches and remains at the minimum temperature setpoint (T is smaller than or equal 3 °C) for 30 min. The IoT-BC would send an email and SMS to a designated administrator as an alert if the internal temperature remained smaller than the minimum temperature set point for 30 min after turning the compressor off.
- Compressor Temperature (CT): It is shown in Figure 8. This figure cares about the compressor temperature. The compressor will be turned off if its temperature reaches the maximum compressor temperature setpoint (CT is greater than or equal to 100 °C) for 5 min. For example, suppose the compressor temperature remained greater than or equal to 100 °C for 5 min after turning the compressor off. In that case, the IoT-BC will send an email and SMS to a designated administrator as an alert.
- Current Meters (CM): It is shown in Figure 9. This figure controls the compressor on/off based on the measures of three current meters (CM1, CM2, and CM3). The three current meters must record the normal range of 4 A to 7 A. If the normal range for the three current meters is satisfied, then IoT-BC must make sure that the absolute difference between any of the three current meters is not greater than or equal to 1 A. Thus, the compressor must keep working normally, and there is no problem. However, suppose the normal range condition is true and the differences between any of the three current meters are greater than or equal to 1 A. In that case, IoT-BC will wait for 1 min and then recheck the differences in the current measures. If the differences in the current measures are still greater than or equal to 1 A, the IoT-BC will turn the compressor off and send an email and SMS to a designated administrator as an alert. If the normal range condition is false, IoT-BC will check if any of the three current meters is greater than or equal to 10 A. Then, IoT-BC will wait for 1 min before rechecking the same condition. If the measure for one of the current meters remained greater than or equal to 1 A for one minute, The IoT-BC will turn the compressor off and send an email and SMS to a designated administrator as an alert.

#### 2.4.2. Humidification Unit’s Control and Alert Mechanism

**Figure 10:**
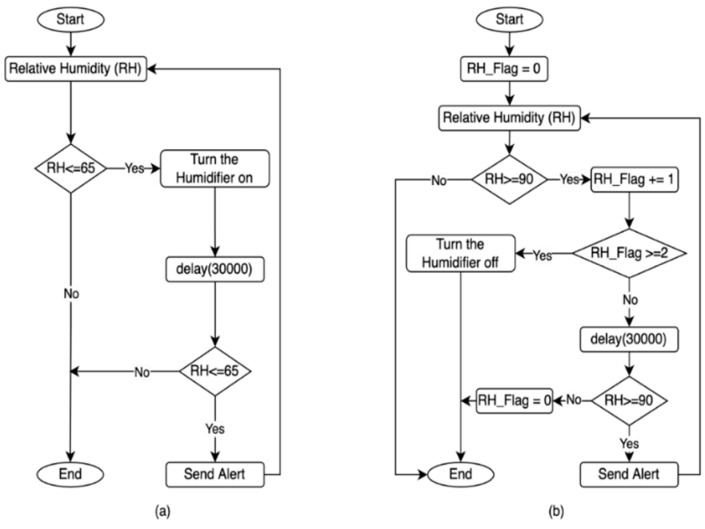
The Humidification unit control and alerts based on the relative humidity (RH) measured data by the DHT22 sensor. (**a**) The control and alert based on the minimum setpoint. (**b**). The control and alert based on the maximum setpoint.

The process of controlling the humidification unit in the MCSR has been accomplished based on the internal relative humidity (RH) measured by the DHT22 sensor, as shown in Figure 10. This figure is composed of two subgraphs:

○ Figure 10a: This flowchart turns the humidifier on when the internal RH reaches the minimum humidity setpoint (RH is smaller than or equal to 65%). The IoT-BC would send an email and SMS to a designated administrator as an alert if the internal humidity remained smaller than the minimum humidity set point for 30 min after turning the humidifier on.
○ Figure 10b: This flowchart turns the humidifier off when the internal humidity reaches and remains at the maximum humidity setpoint (RH is greater than or equal to 90%) for 30 min. The IoT-BC would send an email and SMS to a designated administrator as an alert if the internal humidity remained greater than the maximum humidity set point for 30 min after turning the humidifier off.

#### 2.4.3. Ventilation Unit Control and Alert Mechanism

**Figure 11:**
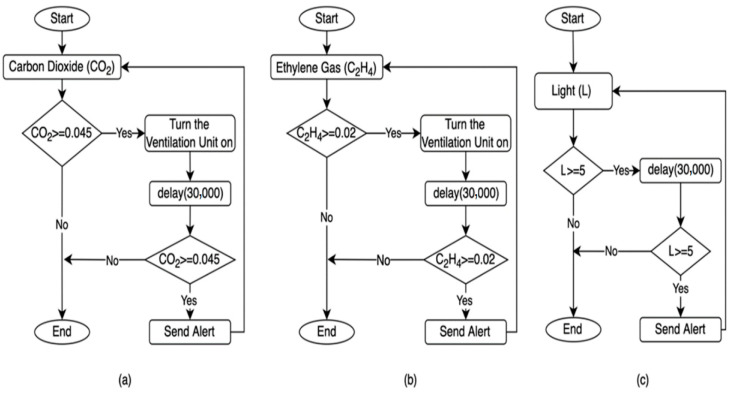
The control and alert based on three measured parameters. (**a**) The ventilation unit control and alerts based on the CO_2_ level measured by the Senseair-S8 sensor. (**b**). The ventilation unit control and alerts are based on the C_2_H_4_ level measured by the MQ-3 sensor. (**c**) The light alert is based on the light level measured by the BH1750 sensor.

The process of controlling the ventilation unit in the MCSR has been accomplished based on two main parameters:

○ Internal Carbon Dioxide (CO_2_): It is shown in Figure 11a. This flowchart turns the ventilation unit on when the internal CO_2_ reaches the maximum CO_2_ Setpoint (CO_2_ is greater than or equal to 0.045%). The IoT-BC would send an email and SMS to a designated administrator to alert if the internal CO_2_ remained greater than the maximum CO_2_ Setpoint for 30 min after turning the ventilation unit on.
○ Internal Ethylene Gas (C_2_H_4_): It is shown in Figure 11b. This flowchart turns the ventilation unit on when the internal C_2_H_4_ reaches the maximum C_2_H_4_ Setpoint (C_2_H_4_ is greater than or equal to 0.02%). The IoT-BC would send an email and SMS to a designated administrator as an alert if the internal C_2_H_4_ remained greater than the maximum C_2_H_4_ set point for 30 min after turning the ventilation unit on.

#### 2.4.4. Light Alert Mechanism

The IoT-BC will send an email and SMS to a designated administrator as an alert if the internal light remains greater than the maximum light set point (L is greater than or equal to 5 Lux) for 30 min, as shown in Figure 11c.

### 2.5. Energy Consumption

The voltage, current intensity, and power factor for TCSR were measured using a real RMS digital power and harmonics clamp meter (UNI-T UT243, Sinotronics Co., Ltd., Guizhou, China). The clamp meters were wired to a laptop for real-time data logging. The following equations were used to estimate the power and energy consumption:

(1) $$P_{1}=V_{\left(L-N\right)}\times I\times P_{F}$$

(2) $$P_{2}=\sqrt{3}\times V_{\left(L-L\right)}\times I\times P_{F}$$

(3) $$E=\left(P_{1}\times T_{1}\right)+\left(P_{2}\times T_{2}\right)$$

where $P_{1}$ is the actual power consumption for a single-phase, $P_{2}$ is the actual power consumption for 3 phases, $V_{\left(L-N\right)}$ is the voltage (line to neutral), $V_{\left(L-L\right)}$ is the voltage (line to line), I is the current intensity, and $P_{F}$ is the power factor ($P_{F}=\cos\varphi )$, $P_{F\;}≃\;0.94$ in the experimental site, E is the total energy consumption, and T_1_ and T_2_ are the actual operating time.

### 2.6. Tested Date Fruits

The yield of thirty date palm trees (Khalas cv.) were selected for this study from the experimental farms of the DPRC at the Agricultural Training and Research Station, KFU, SA (Latitude: 25.267690° N, Longitude: 49.708162° E). All selected date palm trees were almost the same age (16 years) and uniform in growth and were subjected to the same agricultural treatments. The fruits of these palm trees were harvested at a fully ripened stage (Tamr). Immediately after harvesting, the fruits with good characteristics and free from insect damage were transported to the postharvest laboratory at the DPRC. The fruits were sorted and cleaned using dry air and then divided into two similar groups (The total weight was 800 kg). The fruits were packaged in 40 plastic crates with an internal size of 50 × 40 × 25 cm. Each crate has been filled with approximately 20 kg of date fruit. The crates were stored immediately after packing in the two Cold Storage Rooms CSRs, i.e., MCSR and TCSR. The MCSR and TCSR have the same storage temperature (5 °C), same size (482), and manufacturing materials.

### 2.7. Fruit Quality Measurements

The most important quality parameters of the stored date fruits were measured before and after cold storage to study the impact of a controlled atmosphere on fruit quality. Therefore, the following physicochemical characteristics were measured immediately after harvesting at the Tamar stage (full ripened stage with brown color) and during cold storage use. The tested fruits were randomly selected before and during cold storage from different locations in the MCSR and TCSR.

The fruit weights were measured using a digital balance (MSA225S, Sartorius Lab Instruments GmbH Co, Göttingen, Lower Saxony, Germany). Fruit length (FL) and diameter (FD) were measured using a digital vernier caliper. For determining the percentage of fruit weight loss, the fruit samples were weighed before cold storage to obtain the initial weight and after 1, 2, and 3 months to obtain the weight after cold storage. The following equation was utilized to calculate the fruit weight loss:

(4) $$\mathrm{FWL}=\frac{W_{1}-W_{2}}{W_{1}}\times 100$$

where FWL is the fruit weight loss (%), W_1_ is the initial weight of the sample (g), W_2_ is the weight of the same date sample after the target cold storage time (g).

The projected area (PA) of fruits was measured based on the image processing method. A digital camera was used with a fixed height of 50 cm, and a light-emitting diode (LED) source with a power of 20 W was used to adjust the lighting. First, the captured images were transferred to a laptop via a USB port; then, the images were processed using the open-source image-processing software (ImageJ/Fiji 1.46) to determine the required parameters [39].

The fruit volume (FV) was estimated using the gas displacement method in an isothermal system with a pressure of 200 kPa and a temperature of 22 °C. This method used a cylinder with compressed air, an empty cylinder, and a treatment chamber made from stainless steel with 0.01 cm thickness, a length of 100 cm, and an outer diameter of 40 cm [40].

The fruit density (FD) was determined by dividing the mass of the sample by its volume, which was determined by the gas displacement methods. While the bulk density of dates was determined by filling a container of 5000 cm^3^ volume with a date sample, the date sample was weighted to measure its mass. The bulk density of the sample was calculated by dividing the measured mass by 5000 cm^3^.

Fruit sphericity of was defined as the ratio of fruit volume to the sphere with a diameter equal to the major length of the fruit. The fruit sphericity was determined using the following equation [41]:

(5) $$\Phi =\frac{\sqrt[{3}]{l\times w\times t}}{l}$$

where Φ is fruit sphericity, l, w, and t are the length, width, and thickness of the tested fruit (mm), respectively.

Fruit hardness was measured using a texture profile analyzer meter (TA.XTplus, Stable Micro Systems Ltd., Godalming, Surrey, UK) with a cylindrical puncture probe with a 7 mm diameter at room temperature (about 22 °C). The probe traveling speed was 0.5 mm/s, and the puncture distance was 5 mm. The maximum force was recorded during the punching process to indicate the hardness of the stored date fruits.

The moisture content of fruits was determined by drying a sample of 150 g at 70 °C under a vacuum for 48 h using the vacuum-drying oven (LVO-2041P, Daihan Labtech Co., Ltd., Namyangju-si, Gyeonggi-do, Korea) according to the standard methods of analysis of AOAC [42].

The pH, total soluble solids (TSS), moisture content (MC), and water activity (Aw) of the stored fruits were measured according to standard AOAC analysis methods [42]. The laboratory pH meter (HI-99121, Hanna Instruments, Leighton Buzzard, Bedfordshire, UK) was used to determine the pH data. The laboratory refractometer (RFM 860, Bellingham & Stanley Ltd., Kent, UK) was used to determine the TSS data. The portable water activity device (Aqualab Series 3, Decagon Devices, Inc., Pullman, DC, USA) Aw was used to determine the Aw data. A portable electronic moisture balance (Model MOC-120H, Shimadzu Corporation, Kyoto, Japan) was used to determine the MC data.

The color parameters of the fruits were determined based on CIELAB color space using a Hunter Lab color meter (Quest-45/0 LAV, Hunter Associates Laboratory Inc., Reston, VA, USA). The color parameters were measured for each trial using 10 date fruits selected randomly. Chroma, hue angle, and the color difference between the measuring color parameters of fruit before and after storage time were calculated using the following equations:

(6) $$C^{2}=a^{2}+b^{2}$$

(7) $$C=\sqrt{a^{2}+b^{2}\;}$$

(8) h°=arc tan(ba) if the value ≥0

(9) h°=arc tan(ba)+360 otherwise

(10) $$\Delta E=\sqrt{{\left(L_{2}-L_{1}\right)}^{2}+{\left(a_{2}-a_{1}\right)}^{2}+{\left(b_{2}-b_{1}\right)}^{2}}$$

where L is the lightness, a is the red/green coordinate, b is the yellow/blue coordinate, C is Chroma, h° is Hue angle (degree), a is redness, b is yellowness, and ΔE is a color difference.

### 2.8. Statistical Analysis

The statistical metrics were utilized to validate the accuracy of the used sensors by comparing the measured values by the sensors with the reference value simultaneously using standard instruments. These metrics included the determination coefficient, root mean square error, and index of agreement. The determination coefficient (R-squared correlation (R^2^) expresses the strength of the correlation between the measured data and the observed data. The following formula expresses the R-value:

(11) $$R=\frac{N\;(\sum \left(\mathrm{XY}\right)-\left(\sum X\right)(\sum Y)}{\sqrt{\left(n\sum X^{2}-{\left(\sum X\right)}^{2}\right)\left(n\sum Y^{2}-{\left(\sum Y\right)}^{2}\right)}}$$

where R is Pearson correlation, N is the number of data points, X is the observed data, and Y is the measured data.

Root mean square error (RMSE) compares the difference between measured values by the sensor and the observed values measured by the standard instrument. The following formula defines the RMSE:

(12) $$\mathrm{RMSE}=\sqrt{\frac{\sum_{i=1}^{n}{\left(X_{i}-Y_{i}\right)}^{2}}{N}}$$

where RMSE is the root mean square error, X_i_ is the observed data, Y_i_ is the measured data, and N is the number of data points.

The index of agreement (IOA) describes the ratio of the mean square error and the potential error. The IOA varies between 0 and 1, which the agreement value of 0 indicating no agreement at all and 1 indicating a perfect match.

(13) $$\begin{array}{c} \mathrm{IOA}=1-\frac{\sum_{i=1}^{n}{\left(X_{i}-Y_{i}\right)}^{2}}{\sum_{i=1}^{n}{\left(\left|Y_{i}-{\bar{X}}_{o}\right|+\left|X_{i}-{\bar{X}}_{o}\right|\right)}^{2}}\;\\ 0\leq d\leq 1 \end{array}$$

where IOA is the index of agreement, X_o_ is the observed data, Y_m_ is the measured data, ${\bar{X}}_{o}$ is the average data observed.

The statistical analyses of the data were conducted at a 0.05 significance level by the one-way analysis of variance ANOVA using the statistical analysis program of IBM SPSS version 24 (SPSS Inc., Chicago, IL, USA). In addition, the Tukey test was used to determine the least significant difference (LSD) between the experimental mean values at a 0.05 probability level.

## 3. Results and Discussion

### 3.1. Sensors Calibration

Some environmental factors, i.e., high-temperature levels, abnormal humidity conditions, sudden degradation, shocks, etc., can affect the accuracy of the measured values for the sensors used in MCSR. These factors let the sensors give inadequate measurements. Therefore, there is an urgent need to apply sensor calibrations that are subject to most of the mentioned factors to validate sensor measurements before use. In addition, the output signals of the sensors need to calibrate against the reference standard instruments for producing calibration curves that describe the responses of the sensors to the reference instrument data to ensure data acquisition quality [43,44].

Before deploying the sensors for the MCSR measurements, the sensors were calibrated against the suitable reference standard instruments. Table 1 shows the comparison between the measured values and the reference values for temperature, RH, carbon dioxide (CO_2_), ethylene gas sensor (C_2_H_4_), light intensity, current intensity, power, and energy consumption using the standard statistical metrics of the Pearson correlation (R), determination coefficient (R^2^), root mean square error (RMSE), and index of agreement (IOA). This table shows that the calibration of the used sensors achieved the required accuracy within the target parameters based on the R, R^2^, RMSE, and IOA, which had acceptable values for the used sensors.

**Figure 12:**
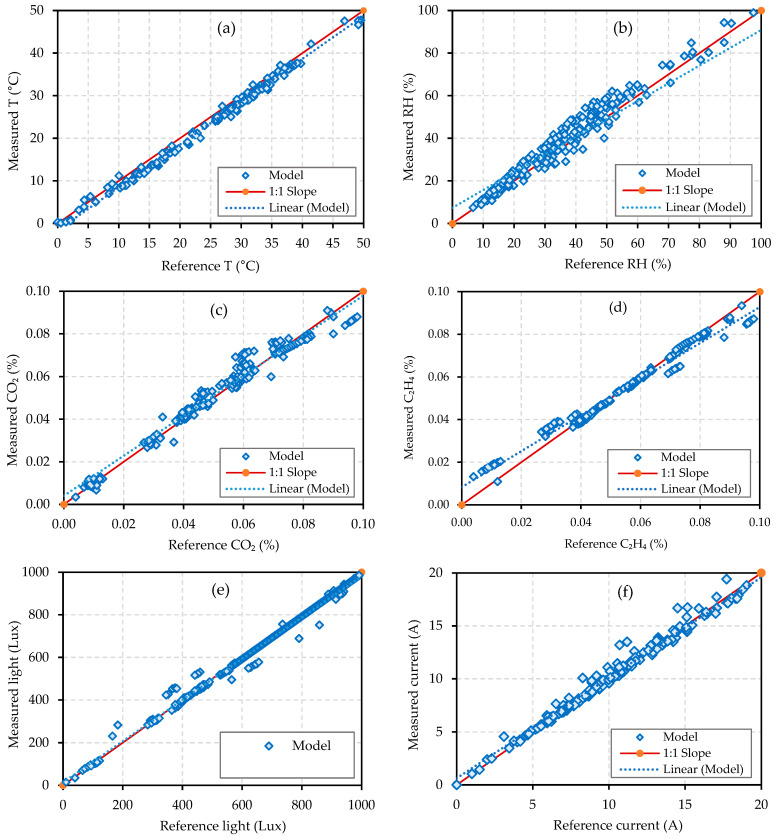
Calibration results for temperature (**a**), relative humidity (**b**), CO_2_ (**c**), C_2_H_4_ (**d**), ambient light (**e**), and current (**f**).

The sensor calibrations have been applied for six sensors in IoT-BC, as shown in Figure 12 and Table 1. The observed values are plotted on the x-axis, and the measured values are plotted on the y-axis for each calibration curve shown in Figure 6. The details of each calibration curve are as follows:

- Figure 12a,b: These curves indicate the temperature and RH calibration. The measured temperature and RH using the DHT22 sensor have been validated with the observed measurements by the incubator. The R, R2, RMSE, and IOA are shown on the temperature and RH rows in Table 1, indicating a perfect match in the measured temperature and RH with the observed temperature and RH. The DHT22 sensors showed good performance at the various temperature and RH values and had good linear regressions that nearly overlapped the 1:1 line (y = x + 0). The linear regression was y = 1.0065x − 1.5962 for the temperature and was y = 0.8346x + 7.3535 for the RH.
- Figure 12c: The CO_2_ measurements have been validated in this curve. The measured CO_2_ using the Senseair-S8 sensor was validated with the indoor air CO_2_ m/datalogger (Extech EA80). The values of R, R2, RMSE, and IOA are shown on the CO_2_ row in Table 1, indicating a perfect match between the measured CO_2_ and the observed CO_2_. The Senseair-S8 sensors showed good performance at the various gas concentrations and had good linear regressions (y = 0.9389x + 0.004) that nearly overlapped the 1:1 line.
- Figure 12d: The C_2_H_4_ measurements have been validated in this curve. The measured C_2_H_4_ using the MQ-3 sensor has been validated with the observed measurements by a fruit ripening gas box containing 99.95% pure ethylene. The values of R, R^2^, RMSE, and IOA are shown on the C_2_H_4_ row in Table 1, indicating a perfect match between the measured C_2_H_4_ and the observed C_2_H_4_. The MQ-3 sensors showed good performance at the various gas concentrations and had good linear regressions (y = 0.8445x + 0.0083) that nearly overlapped the 1:1 line.
- Figure 12e: This curve has validated the light measurements. The measured light using the BH1750 sensor has been validated with the observed measurements by the light intensity meter/datalogger (Extech EA33). The values of R, R2, RMSE, and IOA is shown on the light row in Table 1, indicating a perfect match between the measured light and the observed light. The BH1750 sensors showed good performance at the various light intensities and had good linear regressions (y = 0.9673x + 13.487) that nearly overlapped the 1:1 line.
- Figure 12f: This curve has validated the current intensity. The measured current intensity using the CST2 sensor has been validated with the observed measurements by the three-phase power and harmonics clamp meter (UNI-T). The R, R^2^, RMSE, and IOA are shown on the current, power, and energy rows in Table 1, indicating a perfect match between the measured current intensity and the observed current intensity. The CST2 sensors showed good performance at the various current intensities and had good linear regressions (y = 0.9481x + 0.632) that nearly overlapped the 1:1 line.

### 3.2. Monitoring, Controlling, and Alerting Results

**Figure 13:**
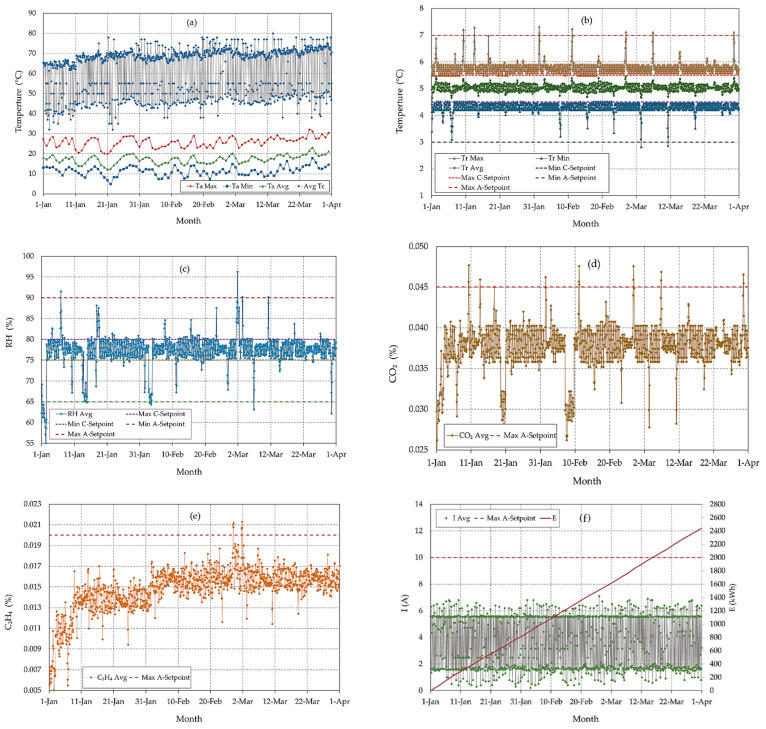
Data acquired by the designed IoT-BC system. Figure (**a**) shows the maximum (Ta Max), minimum (Ta Min), and average (Ta Avg) of the ambient temperature outside. Figure (**b**) shows the maximum (Tc Max), minimum (Ta Min), and average (Ta Avg) of the temperature inside the cold storage room. Figure (**c**) shows the average relative humidity (RH Avg) inside the cold storage room. Figure (**d**) shows the average CO_2_ concentration (CO_2_ Avg) inside the cold storage room. Figure (**e**). Shows the average C_2_H_4_ concentration (C_2_H_4_ Avg) inside the cold storage room and the maximum alert setpoint (Max A-Setpoint). Figure (**f**). Shows the average applied current (I Avg) and the electrical energy consumption (E).

Figure 13 presents the real-time acquired data based on the sensors’ measurements for the most important parameters, i.e., outside temperature and compressor temperature (a), MCSR temperature (b), MCSR RH (c), CO_2_ concentration in the MCSR (d), C_2_H_4_ concentration in the MCSR (e), and applied electrical current and hourly energy consumption (f) for 3 months from 1 January to 1 April 2022.

Figure 13a shows the variations in the maximum temperature (Ta Max), minimum temperature (Ta Min), and average temperature (Ta Avg) temperature for the outside atmosphere in the study area and the average temperature of the MCSR compressor (Tc Avg). The outside temperature ranged from 12.13 to 32.03 °C and the average outside temperature was 20.32 ± 5.09. The average compressor temperature was 62.34 ± 10.91 ranging from 32 to 80 °C. From the collected data in this figure, it is obvious that the outside temperature impacts the compressor temperature. There was no significant difference between the MCSR and TCSR compressor temperatures. The compressor of the MCSR has not automatically turned off, and the designed IoT-based system has not sent any email or SMS as an alert because the temperature has not reached to maximum compressor temperature Setpoint (100 °C for 5 min), as shown in Figure 8.

Figure 13b shows the maximum inside temperature (Tc Max) the minimum temperature (Tc), and the average temperature (Tc Avg) in the MCSR. Each value in this curve describes the average temperatures of three different locations in the MCSR. This temperature was controlled based on the flowchart shown in Figure 7, where Min C-Setpoint is the minimum control setpoint equal to 4.5 °C, and Min A-setpoint is the minimum alert setpoint equal to 3 °C. The Max C-Setpoint and Max A-Setpoint are the maximum control and alert Setpoint that equal 5.5 °C and 7 °C, respectively. Figure 13b assures that the developed MCSR successfully achieved the target internal temperature ranges as controlled and alerted based on the setpoints, as shown in Figure 7. Unlike the TCSR the designed system successfully sent an alert when the CCAR temperature reached 7.21 °C on 10 January at 8:00, 7.30 °C on 13 January at 14:15, 7.32 °C on 1 February at 12:10, 7.24 °C on 11 February at 4:16, 7.12 °C on 26 February at 22:10, 7.11 °C on 6 March at 20:15, and 7.11 °C on 30 March at 14:05 based on the Tc Max A-Setpoint (7 °C). The system also successfully sent an alert when the internal de-creased to 2.82 °C on 3 March at 10:15 and 2.87 °C on 11 March at 06:05 based on the Tc Min A-Setpoint (3 °C).

Figure 13c shows the average RH inside the MCSR for three months. The internal RH in MCSR is controlled based on the flowchart shown in Figure 10, where RH Avg is the average RH inside the cold storage room, Min C-Setpoint is the minimum control setpoint (75%), Max C-Setpoint is the maximum control setpoint (80%), Min A-Setpoint is the minimum alert setpoint that equals 65%, and Max A-Setpoint is the maximum alert setpoint that equals 90%. The control of the humidification system in the MCSR achieved the target RH ranges, as observed in Figure 10. There was a significant difference between the RH in the MCSR and TCSR. Where a significant oscillation in the RH in the TCSR was observed with the passage of storage time. Figure 13c assures that the designed IoT-BC has successfully achieved the target internal RH ranges as controlled and alerted based on the setpoints, as shown in Figure 10. The system successfully sent an alert when the average RH reached 91.53% on 6 January at 22:00 and 91% on 1 March at 20:05 based on the RH Max A-Setpoint (90%). The system also successfully sent an alert when the average RH decreased to 64.38% on 1 January at 06:00, 63.07% on 1 January at 18:07, 64.72% on 3 February at 02:10, 64.35% on 3 February at 12:00, 63.21% on 6 March at 20:10, and 62.21% on 30 March at 14:20, according to the RH Min A-Setpoint (65%).

Figure 13d shows the average CO_2_ concentration (Avg CO_2_) inside the cold storage room. The CO_2_ concentration inside the cold storage was controlled and alerted based on the flowchart shown in Figure 11a, where Max A-Setpoint is the maximum control and alert Setpoint that equals 0.045%. Figure 13d assures that the designed IoT-BC has successfully achieved the target internal CO_2_ ranges as controlled and alerted based on the setpoints, as shown in Figure 11a. Unlike the TCSR, the designed system successfully sent an alert when the average CO_2_ concentration reached to 0.0477% on 10 January at 08:00, 0.0459% on 13 January at 14:00, 0.0462% on 1 February at 12:10, 0.0476% on 11 February at 4:15, 0.0476% on 26 February at 22:02, 0.0469% on 6 March at 22:05, and 0.0465% on 30 March at 14:15 based on the CO_2_ Max A-Setpoint (0.045%).

Figure 13e shows the average C_2_H_4_ concentration (C_2_H_4_ Avg) inside the cold storage room. The C_2_H_4_ concentration inside the cold storage room was controlled and alerted based on the flowchart shown in Figure 11b, where Max A-Setpoint is the maximum control and alert Setpoint of 0.02%. Figure 13e assures that the designed IoT-BC has successfully achieved the target internal C_2_H_4_ ranges as controlled and alerted based on the setpoints, as shown in Figure 11b. The system successfully sent an alert when the average C_2_H_4_ concentration reached 0.0212% on 27 February at 04:10 and 0.0213% on 1 March at 20:15 based on the C_2_H_4_ Max A-Setpoint (0.02%).

Figure 13f shows the average required current (I Avg) and the hourly electrical energy consumption for the MCSR equipment. The electrical energy is consumed by the refrigeration of date fruits, evaporator, and condenser fans, defrost heaters, humidification system, lights, and control system. The average current for the cold storage room is monitored based on the flowchart shown in Figure 9, where Max A-Setpoint is the maximum control and alert Setpoint that equals 10 A. Figure 13f assures that the developed IoT-BC has successfully achieved the current target ranges as controlled based on the setpoints, as shown in Figure 9. There was no significant difference between the MCSR and TCSR applied electrical energy consumption although the MCSR was supplied with the designed control and humidification systems. The MCSR compressor has not automatically turned off and the designed IoT-based system has not sent any email or SMS as an alert because the applied electrical current has not reached to maximum current Setpoint (10 A for one minute) and the three current meters of the compressor recorded the normal ranges from 4 A to 7 A during its operating, as shown in Figure 9.

Inanition, the designed IoT-based system has not sent any email and SMS alerts due to the internal light has not remained greater than the maximum light Setpoint (L is greater than or equal to 5 Lux) for 30 min, as shown in Figure 11c.

### 3.3. Impact of IoT-BC on Stored Fruit Characteristics

Table 2 displays the impact of controlling and monitoring the MCSR by IoT-BC under controlled RH of 75–80%, 5 °C temperature, and CO_2_ and C_2_H_4_ gases on the most important quality parameters, i.e., fruit weight, weight loss percentage, length, diameter (FD), projected area, volume, density, sphericity percentage, hardness, pH, total soluble solids, moisture content, water activity, color parameters, and color difference of stored date fruits (Khalas cv.) compared to the TCSR under a same controlled temperature of 5 °C during cold storage times.

The data in Table 2 show a highly significant (*p* < 0.05) effect of the storage time on the most quality parameters of stored date fruit in the TCSR compared with the MCSR. Comparing TCSR and MCSR after 3 months of storage time, date palm fruits stored in TCSR had significantly (*p* < 0.05) lower fruit length, volume, hardness, pH, and color parameters (b, h, and c). The fruit’s total soluble solids, moisture content, water activity, and color lightness (L) were significantly increased when date palm fruits were stored at different duration in the TCSR and the MCSR. There was no significant difference between the mean values of fruit weight, diameter, projected area, density, sphericity, and color parameter (a). There was no significant weight loss and color difference for the stored date fruit in the MCSR. On the contrary, there was a significant difference in weight loss and color difference for dates stored in the TCSR.

Food products must be properly stored in some cold storage facilities to prevent spoilage and save their nutritional value. At least, there should exist an effective control and monitoring for the temperature and humidity inside the cold storage facilities. This motivates the need for integrating IoT with the cold storage facility. The IoT-enabled cold storage monitoring can regularly and successfully record, monitor, and support the conditions inside the cold storage. It is also urgent to ensure that the cold storage room temperature never exceeds the optimal temperature based on the kind of product stored in the room.

The most critical parameters that affect the shelf life of the stored products in cold storage are temperature and RH [45,46]. The fruits of date palm cv. Khalas stored at 5 °C and 80% RH did not show insect activity or degradation in fruit quality and enhanced their shelf-life [47]. Soft and semi-soft date fruits can be preserved by storing them at 5 °C or lower, preventing the development of various pests [48]. However, the stored product is subject to partial weight loss during cold storage in conventional systems due to its surface drying in addition to the deterioration of the quality of the fruits during storage due to the lack of accurate control of the temperature and RH in the internal atmosphere of cold refrigerating rooms.

The morphological and biochemical characteristics of date palm fruit, such as size, color, hardness, acidity, sweetness, and moisture content, are used to assess its marketability [49]. The results found that fruit weight, size, and projected area were maintained better after three months of storage at MCSR, where the RH was remotely controlled. These parameters were significantly reduced at TCSR, where RH oscillated. Previous studies on date palm fruit storage indicated a reduction in fruit weight and size with time [4,50,51]. The management of storage temperature was emphasized in these investigations. However, the current study focused on storage temperature and RH. As a result, the fruits stored at MCSR did not reduce weight or size after three months of storage.

Similarly, the fruit weight loss was also minimal after three months of storage at MCSR compared to TCSR. The increase in respiration rate, induced by reduced RH in the air surrounding the fruit, is usually responsible for the decrease in fruit weight [52,53]. The fruit weight loss was higher with the increase in storage temperature in apples; however, higher RH reduced the weight loss [54]. The ultrasonic humidifier substantially impacted the weight loss of date fruits after six months of cold storage [9]. After three months of storage at MCSR, maintaining the RH retained fruit weight in the current study.

Similarly, the date palm fruit firmness was significantly reduced at TCSR compared to MCSR after six months of storage. Fruit tissue softening is linked to moisture loss and turgor pressure reduction [55]. In the present study, maintaining a high RH level equilibrated moisture content, which kept the fruit firm. Nectarine fruits have reduced weight loss and maintained higher fruit firmness during cold storage under 85–90% RH [56]. On the other hand, apple fruits kept at 95% RH lost their firmness very slowly during storage [54]. Similarly, high RH during storage-maintained firmness in zucchini fruit [57].

However, the fruit pH of date palm cv. Khalas stored at TCSR or MCSR was reduced after three months of storage; however, it was lowest at TCSR. The fermentation activity of microorganisms during storage is considered to produce organic acids, leading to decreased fruit pH [58,59]. Similar results were reported after fruit storage in cv. Khalas [16] and cv. Stamaran [60]. According to the present study, the TSS of date palm cv. Khalas stored for three months increased at both storage conditions, i.e., 15.96% at TCSR and 10.88% at MCSR. The leading cause of the increase in TSS would be the enzymatic conversion of large polysaccharides into small sugars [4]. According to Radi et al. [59], the increase in TSS of date fruits during storage may be due to microbial and enzymatic activity that converts high molecular weight molecules to low molecular weight compounds. Pomegranate fruit had a considerable rise in TSS content during storage, linked to moisture loss and increased sugar concentration in the fruit [61,62]. These results coincide with Aleid et al. [63], who stored date fruits (cv. Khalas) at 5 °C for 12 months and found that fruit pH declined while TSS and moisture content increased. Similarly, TSS increased in cv. Mazafati fruits are stored at 4 °C for 180 days [51]. Pomegranate fruits stored at 5 °C and 92% RH had significantly reduced weight loss and maintained TSS [64]. Our study also showed that the fruits were stored at TCSR and MCSR for three months, and the moisture content of the fruit increased by 30.85 and 18.85%, respectively, compared to the fruits before storage. Comparing the moisture content of fruits stored at TCSR and MCSR after three months, 16% less moisture content was determined in fruit stored at MCSR. Similarly, water activity was 14.44% less after three months of storage at MCSR compared to TCSR. Mohammed et al. [9] found that fruits of cv. Khalas stored at 5 °C and 80% RH maintained moisture content and water activity compared to traditional storage facilities. Another study revealed that after five months of storage, the moisture content in cvs. Majhoul and Boufeggous did not change considerably, whereas TSS increased in cv. Majhoul [65]. The decrease in moisture and water activity could be accounted for by the evaporation of fruit water caused by the relatively high temperature and moderate RH [66].

Apart from the values of CIE L, all other fruit color attributes (a, b, h°, and C) decreased during the storage time; however, these attributes (a, b, h°, and C) were significantly higher at MCSR when compared with TCSR color values. The decrease in h° was also reported in tomatoes during storage [67]. In cherries, the h° decreased under both cold and ambient temperature storage [68]. In the present study, the h° difference between unstored and stored fruits for three months at MCSR was non-significant, also reported in strawberries where storage temperature and RH did not affect h° [69]. A significant difference in color attributes was also observed in apple cv. Granny Smiths were stored for 4 months at 2 °C and 90% RH [45]. On the other hand, pomegranate fruits stored at 5 °C maintained their color better throughout the storage period [64].

## 4. Conclusions

Maintaining high quality and ensuring food safety are extremely important. The present study presented a smart IoT-BC system that connects sensors, actuators, and related cold storage equipment for remote controlling, monitoring, and risk alerting to maintain food quality and components of cold storage facilities. As a case study, we applied the cloud-based IoT system to control and monitor the microclimate, i.e., RH, temperature, CO_2_, C_2_H_4_, and interior light for a commercial-sized CSR, for extending the shelf life and maintaining the quality of the date fruit (Khalas cv.). On the other hand, some operating parameters, i.e., the temperature of the refrigeration compressor, the electrical current, and the energy consumption, were monitored and controlled to maintain CSR equipment. The impacts of the designed IoT-based control system on date fruit quality during cold storage in the MCSR were investigated and compared with TCSR. Based on real-time data analytics, the results showed that the designed IoT-BC system efficiently controlled the MCSR, provided reliable data about the interior microclimate atmosphere, applied electrical current and energy consumption of the MCSR, and sent the necessary alerts in the event of an emergency. In the MCSR, most of the quality attributes did not significantly differ at various storage times; however, in the TCSR, storage time had an adverse effect on the quality characteristics of stored date fruit. Therefore, we recommend IoT-BC technology to manage cold storage facilities to maintain the high quality and safety of the stored food due to its positive impacts on the characteristics of stored fruits and the potential of remote control and monitoring of cold storage facilities. However, further studies are needed to incorporate the designed IoT-BC with modern machine learning and food sensor technologies for integrated food quality management during cold storage.

## Figures and Tables

**Table 1:** Statistical metrics values of Pearson correlation (R), determination coefficient (R^2^), root mean square error (RMSE), and index of agreement (IOA)resulting from comparing the measured values of the temperature and RH sensor (DHT22), carbon dioxide (CO_2_) sensor (Senseair-S8), ethylene gas (C_2_H_4_) sensor (MQ-3), light intensity sensor (BH1750), current intensity sensor (CST2), calculated power, and calculated electrical energy consumption with the reference values.

Parameters	Statistical Metrics			
	R	R^2^	RMSE	IOA
Temperature	0.997	0.994	1.673	0.995
RH	0.867	0.752	10.18	0.927
CO_2_	0.983	0.966	0.004	0.99
C_2_H_4_	0.991	0.982	0.004	0.989
Light	0.995	0.990	26.05	0.997
Current	0.997	0.994	0.801	0.998
Power	0.997	0.993	175.3	0.998
Energy	0.996	0.992	257.2	0.997

The numbers of the measured points (n) were 200 values for all calibrated parameters.

**Table 2:** Comparison of mean values ± standard deviation of fruit weight (FW), fruit weight loss (FWL), fruit length (FL), fruit diameter (FD), projected area (PA), fruit volume (FV), fruit density (Fd), sphericity percentage (Φ), fruit hardness (FH), pH, total soluble solids (TSS), moisture content (MC), water activity (Aw), color parameters (L, a, b, h, c), and color difference (∆E) of stored date fruits (Khalas cv.) at various storage time in cold storage rooms (CSRs) under traditional cold storage room (TCSR) and the modified cold storage room (MCSR) controlled by IoT- based control system.

Characteristics	CSRs	Storage Time (Months)			
		0	1	2	3
FW (g)	TCSR	11.23 ± 1.3 ^A^	10.69 ± 1.13 ^AB^	9.63 ± 1.02 ^B^	11.01 ± 0.07 ^AB^
	MCSR	11.29 ± 0.76 ^A^	11.16 ± 0.88 ^A^	10.63 ± 0.68 ^AB^	11.32 ± 1.02 ^A^
FWL (%)	TCSR	0 ± 0	5.77 ± 0.17 ^B^	19.61 ± 0.59 ^A^	4.81 ± 1.34 ^C^
	MCSR	0 ± 0	1.35 ± 0.09 ^D^	1.96 ± 0.14 ^D^	1.75 ± 0.26 ^D^
FL (mm)	TCSR	45.43 ± 2.11 ^A^	44.07 ± 1.84 ^AB^	44.13 ± 3.19 ^AB^	41.37 ± 2.27 ^B^
	MCSR	44.7 ± 2.02 ^A^	44.38 ± 1.83 ^A^	43.04 ± 1.7 ^AB^	44.41 ± 1.48 ^A^
FD (mm)	TCSR	25.72 ± 0.9 ^A^	25.04 ± 0.7 ^A^	24.35 ± 1.37 ^A^	24.64 ± 1.05 ^A^
	MCSR	24.98 ± 2.27 ^A^	25.01 ± 1.17 ^A^	24.33 ± 1.37 ^A^	24.81 ± 1.36 ^A^
PA (cm^2^)	TCSR	6.76 ± 0.33 ^A^	6.38 ± 0.25 ^A^	6.36 ± 0.64 ^A^	6.3 ± 0.6 ^A^
	MCSR	7 ± 0.87 ^A^	6.69 ± 0.33 ^A^	6.33 ± 0.48 ^A^	6.65 ± 0.54 ^A^
FV (cm^3^)	TCSR	15.39 ± 1.08 ^AB^	14.15 ± 0.78 ^AB^	13.77 ± 1.75 ^B^	11.94 ± 1.9 ^C^
	MCSR	15.63 ± 1.13 ^A^	14.89 ± 0.44 ^AB^	13.79 ± 1.05 ^B^	14.7 ± 0.99 ^AB^
Fd (g/cm^3^)	TCSR	0.93 ± 0.1 ^A^	0.95 ± 0.11 ^A^	1.01 ± 0.18 ^A^	1.02 ± 0.14 ^A^
	MCSR	0.95 ± 0.1 ^A^	0.95 ± 0.07 ^A^	0.98 ± 0.09 ^A^	0.99 ± 0.09 ^A^
Φ (%)	TCSR	82.89 ± 3.63 ^A^	83.08 ± 3.24 ^A^	83.5 ± 5.54 ^A^	82.21 ± 3.81 ^A^
	MCSR	82.17 ± 3.25 ^A^	82.62 ± 1.5 ^A^	82.83 ± 2.81 ^A^	82.19 ± 2.74 ^A^
FH (N)	TCSR	4.94 ± 1.87 ^AB^	3.63 ± 1.28 ^BC^	2.07 ± 0.61 ^DE^	1.33 ± 0.67 ^E^
	MCSR	5.06 ± 0.8 ^A^	4.34 ± 0.66 ^A–C^	3.03 ± 0.23 ^CD^	3.19 ± 0.34 ^CD^
pH	TCSR	8.36 ± 0.07 ^AB^	8.12 ± 0.07 ^BC^	7.65 ± 0.13 ^E^	7.15 ± 0.02 ^F^
	MCSR	8.48 ± 0.4 ^A^	8.3 ± 0.19 ^A–C^	8.06 ± 0.22 ^CD^	7.81 ± 0.19 ^DE^
TSS (°Brix)	TCSR	74.57 ± 1.06 ^D^	75.54 ± 0.68 ^CD^	77.5 ± 0.68 ^C^	88.73 ± 0.97 ^A^
	MCSR	73.84 ± 2.44 ^D^	74.69 ± 1.24 ^D^	75.67 ± 0.9 ^CD^	82.85 ± 3.27 ^B^
MC (%)	TCSR	19.88 ± 0.72 ^F^	22.01 ± 0.8 ^DE^	26.28 ± 1.38 ^B^	28.75 ± 1.39 ^A^
	MCSR	19.63 ± 0.66 ^F^	20.82 ± 0.61 ^EF^	22.96 ± 0.83 ^CD^	24.19 ± 0.75 ^C^
Aw	TCSR	0.62 ± 0.03 ^E^	0.66 ± 0.03 ^DE^	0.76 ± 0.06 ^BC^	0.9 ± 0.05 ^A^
	MCSR	0.64 ± 0.08 ^DE^	0.65 ± 0.04 ^DE^	0.7 ± 0.04 ^CD^	0.77 ± 0.05 ^B^
L	TCSR	43 ± 2.84 ^D^	44.74 ± 1.99 ^CD^	48.21 ± 2.1 ^B^	51.72 ± 1.4 ^A^
	MCSR	43.73 ± 3.43 ^D^	44.23 ± 1.65 ^D^	45.97 ± 1.85 ^BD^	47.72 ± 1.08 ^BC^
a	TCSR	9.83 ± 2.73 ^A^	9.64 ± 1.7 ^A^	9.26 ± 3.1 ^A^	4.99 ± 1.24 ^B^
	MCSR	9.58 ± 1.79 ^A^	9.61 ± 0.82 ^A^	9.42 ± 1.46 ^A^	7.29 ± 0.81 ^AB^
b	TCSR	13.05 ± 1.63 ^A^	11.4 ± 1.22 ^A–C^	8.08 ± 2.94 ^DE^	6.71 ± 1.37 ^E^
	MCSR	12.81 ± 1.23 ^A^	12.1 ± 0.57 ^AB^	10.45 ± 1.37 ^BC^	9.76 ± 0.86 ^CD^
h°	TCSR	64.94 ± 5.56 ^A^	60.51 ± 3.62 ^AB^	64.79 ± 5.19 ^A^	50.6 ± 4.39 ^C^
	MCSR	66.15 ± 5.69 ^A^	63.33 ± 2.49 ^AB^	57.8 ± 3.06 ^B^	65.47 ± 3.37 ^A^
c	TCSR	16.39 ± 2.89 ^A^	14.94 ± 1.95 ^A–C^	12.31 ± 4.2 ^C^	8.38 ± 1.74 ^D^
	MCSR	16.87 ± 1.77 ^A^	15.91 ± 1.44 ^AB^	14.59 ± 2.49 ^A–C^	12.63 ± 1 ^BC^
∆E	TCSR	0 ± 0	3.21 ± 0.78 ^C^	6.43 ± 1.56 ^AB^	7.14 ± 2.75 ^A^
	MCSR	0 ± 0	2.65 ± 2.34 ^C^	3.51 ± 1.36 ^C^	4.4 ± 2.21 ^BC^

The means within each quality parameter (N = 10) with the same letters are not significantly different at *p* ≤ 0.05.

## Data Availability

Not applicable.
